# The Relationship between the UPPS-P Impulsivity Dimensions and Nonsuicidal Self-Injury Characteristics in Male and Female High-School Students

**DOI:** 10.1155/2013/654847

**Published:** 2013-01-30

**Authors:** Laurence Claes, Jennifer Muehlenkamp

**Affiliations:** ^1^Faculty of Psychology and Educational Sciences, University of Leuven, P.O. Box 3720, Tiensestraat 102, 3000 Leuven, Belgium; ^2^Psychology Department, University of Wisconsin Eau Claire, WI, USA

## Abstract

The present study investigated the association between nonsuicidal self-injury characteristics, functions, and the UPPS-P impulsivity-related traits in high-school students using self-report questionnaires. More than 17% of the 613 students engaged in at least one type of NSSI behavior. Compared to male students, female students engaged more often in cutting and less in head banging. All NSSI behaviors were significantly related to Negative and Positive Urgency, that is, the tendency to act impulsive in the presence of negative/positive affect. Interactions between different UPPS-P impulsivity dimensions did not increase the percentage of explained variance in the different NSSI behaviors. Furthermore, severe cutting was negatively related to Lack of Premeditation. Different NSSI functions showed differential relationships with the five UPPS-P impulsivity dimensions.

## 1. Introduction

Non-suicidal self-injury refers to socially unaccepted behavior involving deliberate and direct injury to one's own body surface without suicidal intent [1]. Most individuals who self-injure begin to do so during early to middle adolescence [2], with an average age of onset ranging between 12 and 16 years of age [3]. In a large survey of NSSI among American college students, Whitlock and colleagues [4] reported that 15.3% of the students had engaged in NSSI at least once (6.8% in the previous 12 months). No significant gender differences were found for 12-month prevalence of NSSI. However, females engaged in more cutting than males [4], whereas males engaged in more self-battery than females [4]. Most adolescents engage in NSSI for automatic (self) reinforcement (e.g., to stop bad feelings, to punish oneself), although a sizable minority endorse social reinforcement functions as well (e.g., to make others angry) [1, 5]. Despite the consistent endorsement of these functions by those with an NSSI history, very few studies have examined how features such as impulsive personality trait(s) may influence the functions NSSI serves within an individual.

It is often assumed that individuals who self-injure are more impulsive than those who do not self-injure. In support of this notion, there is evidence that adolescents who engage in NSSI are more likely to engage in other impulsive/risky behaviors [6–8], show more Cluster B personality disorder criteria [7, 9, 10], and show higher scores on impulsivity-related traits [6, 7]. On the contrary, other studies found mixed results regarding the link between NSSI and impulsivity. For example, Hawton et al. [11] found that impulsivity distinguished female, and not male, self-injurious adolescents from healthy controls. Other researchers [12–14] found significant differences between adolescents with/out NSSI by means of self-reported impulsivity, but failed to detect between-group differences based on performance-based tasks of impulsivity. One potential reason for the mixed findings is the various conceptualizations and ways to assess impulsivity. Since impulsivity appears to consist of multiple dimensions, using the UPPS model which posits four distinct pathways to impulsive behavior [15] may help clarify the heterogeneous results found for NSSI. In the UPPS model, Negative Urgency measures an individual's tendency to act impulsively when experiencing negative affect. Lack of Perseverance assesses an individual's tendency to give up in the face of boredom, fatigue, or frustration. Lack of Premeditation assesses an individual's tendency to act without consideration of the potential consequences of the behavior. Sensation Seeking refers to an individual's tendency to pursue activities that are exciting and novel. Recent work by Cyders and Smith [16, 17] has identified an additional dimension, Positive Urgency, which assesses an individual's tendency to act impulsively under conditions of heightened positive affect. As far as we know, only two studies have used the UPPS (without the Positive Urgency dimension) to investigate impulsivity in individuals with/without NSSI. Glenn and Klonsky [12] compared college students with/without NSSI on the UPPS Impulsive Behavior Scale. Self-injurious students were best distinguished by Negative Urgency, and to a lesser degree by Lack of Premeditation and Sensation Seeking. Lack of Perseverance predicted more recent and frequent NSSI. Lynam et al. [15] assessed the incremental validity of the four UPPS impulsivity traits, above and beyond borderline personality disorder, in predicting NSSI in a sample of drug abusers. Significant positive associations were found between NSSI and Negative Urgency (*r* = .25), Lack of Premeditation (*r* = .31), and Lack of Perseverance (*r* = .29). The interaction of Negative Urgency × Lack of Premeditation was able to predict NSSI above and beyond the BPD diagnosis, such that the relation between Negative Urgency and NSSI was stronger among those low on Premeditation. 

The aims of the present study are threefold. Firstly, we assess the prevalence of six different NSSI behavior methods as well as gender differences in their prevalence. Secondly, while the existing literature is helping to elucidate the complex relationship between impulsivity traits and NSSI, the research is limited to the original four-dimensional model. Existing research has yet to include the fifth impulsivity-related trait, Positive Urgency, which was a second aim for the present study. We correlated the five impulsivity-related traits (UPPS-P) with the presence/absence of six different NSSI behaviors among male/female college students. Thirdly, we attempt to expand existing research by examining the relationship between the five impulsivity-related traits and the functions of NSSI, which has not been previously explored. 

## 2. Method

### 2.1. Participants

Our original sample consisted of 651 high-school students (10th till 12th grade) from the Flemish speaking part of Belgium. Only those students who provided information about their age and gender and completed the SIQ-TR and the UPPS-P were included in the study, resulting in a sample of 613 high-school students (60.4% females, 39.6% males). The mean age of the sample was 16.38 years (SD = 1.06, range = 14–20 years). Approximately 106 students (17.3%) were endorsed having engaged in at least one NSSI behavior during life-time, a rate consistent with previous research in college samples [4], and high-school samples [18].

### 2.2. Procedure

Five high schools, located in different areas of the Flemish speaking part of Belgium, participated in the study. School directors were approached by the researchers, informed of the study purpose, and all directors approached agreed to participate. School directors distributed a passive informed consent letter to parents via the students one week before the study. 

All students (consent rate of 100%) who were present the day the study was occurring completed questionnaires during regular school hours. Students were provided with an envelope holding assent documents, questionnaires, and a letter with phone numbers and e-mail addresses of professional help centers. After completing the forms, they returned their questionnaires to the researcher in a sealed envelope, who remained in the classrooms while the students completed the questionnaires. The study was approved by the ethical board of the department of psychology affiliated with the first author. Participants were not compensated for participating in the study.

### 2.3. Instruments

Using the Self-Injury Questionnaire-Treatment Related (SIQ-TR) [19], students responded to 6 yes/no items inquiring whether they had ever deliberately injured themselves by means of head banging, scratching, superficial cutting, severe cutting, hitting, and burning oneself. If they had performed at least one act of NSSI, they were to specify the functions which were served by their NSSI on a scale ranging from 1 (*not at all applicable*) to 5 (*very much applicable*) (see Table 2 for item descriptions). The SIQ-TR has been used in previous studies [20] and demonstrates strong reliability and validity for use with youth [19].

The UPPS-P Impulsivity Scale [17] is a 59-item inventory designed to measure five distinct features of impulsive behavior: Negative Urgency, Lack of Perseverance, Lack of Premeditation, Sensation Seeking, and Positive Urgency. The first 4 dimensions were assessed by the original version of the UPPS [21, 22] and the fifth dimension was added based on work of Cyders and colleagues [16, 17]. Each item of the UPPS is rated on a 4-point Likert scale ranging from 1 (strongly agree) to 4 (strongly disagree). The Cronbach's alphas of the five scales in the present study are as follows: Negative Urgency (*α* = .87), Lack of Perseverance (*α* = .82), Lack of Premeditation (*α* = .84), Sensation Seeking (*α* = .85), and Positive Urgency (*α* = .92).

### 2.4. Analyses

To assess the association between gender and the presence/absence of the six different NSSI methods, we used the chi-square test statistic. The associations between the UPPS-P impulsivity dimensions and the presence/absence of the different NSSI methods were assessed with Spearman's rank correlation coefficients. To investigate the association between the functions of NSSI, we performed a factor analysis on the NSSI functions (to reduce the numbers of items) and correlated the UPPS-P dimensions with the NSSI function scale scores.

## 3. Results

Approximately 106 students (17.3%) reported engaging in at least one NSSI behavior during their life-time. Almost 69% used only one method of NSSI, whereas 19.8% used two, 8.5% used three, and 2.8% four or more different methods of NSSI. Superficial cutting (49.1%) was the most prevalent method, followed by scratching (28.3%), severe cutting (25.2%), head banging (24.5%), hitting (15.1%), and burning (3.8%) oneself. Overall, we did not find a significant relationship between gender and the presence/absence of NSSI (*X*_(1)_^2^ = .44, *ns*); 18.1% of the female students engaged in NSSI compared to 16% of the males. However, females (5.7%) reported significantly more severe cutting compared to males (2.5%) (*X*_(1)_^2^ = 3.58,   *P* < 0.05), whereas males (6.6%) reported significantly more head-banging than females (2.7%) (*X*_(1)_^2^ = 5.44,   *P* < 0.05). 

The Spearman's rank correlations between the five UPPS-P dimensions and the presence/absence of 6 different NSSI methods are given in Table 1. The presence/absence of all the different NSSI methods and both Negative and Positive Urgency (i.e., reactivity to both negative and positive emotions) was significantly and positively related. Additionally, severe cutting showed a positive significant correlation with Lack of Premeditation (i.e., act without thinking).

The relationship between NSSI functions and the UPPS-P impulsivity dimensions was also investigated. To reduce the number of functions, an exploratory factor analysis with oblique rotation was conducted with the 12 NSSI functions. Based on the number of eigenvalues ≥1, and items with a factor loading >.40, a five-factor solution was chosen which explained 72.30% of the variance: F1 (avoid/approach destruction; *n* = 3, *α* = .75), F2 (show being strong, *n* = 2, *α* = .66), F3 (avoid tasks/events, *n* = 2,   *α* = .59), F4 (emotion regulation, *n* = 2,   *α* = .55), and F5 (escape from others, reality, and intrusions, *n* = 3,   *α* = .49) (see Table 2). Scale scores were calculated by summing the items on each factor. The emotion regulation function was endorsed at a greater rate than the other functions. Pearson correlations were calculated between the NSSI function scale scales and the UPPS-P dimensions (see Table 2). F1 (avoid/approach destruction) was significantly positively related with Sensation Seeking, F4 (emotion regulation) with Negative Urgency, and F5 (escape from others, reality, and intrusions) with Positive/Negative Urgency, whereas F2 (show being strong) and F3 (avoid tasks/events) were positively related with Lack of Perseverance.

## 4. Discussion

In the present study, we investigated the association between different NSSI behaviors and the UPPS-P impulsivity traits in high-school students. Overall, 17.3% of the 613 students engaged in at least one type of NSSI, and females were more likely to engage in severe cutting compared to males, who were more likely to engage in head-banging. The descriptive features are consistent with existing reports of NSSI and gender-specific features [4]. Also consistent with existing research were the current findings that the presence/absence of NSSI was positively correlated with the traditional UPPS dimensions of Negative Urgency and Lack of Premeditation [12, 13, 15]. However, the current study expanded upon prior research by including the dimension of Positive Urgency and examining the unique relationship between UPPS-P dimensions with specific NSSI behavioral methods. 

The correlations showed that each of the different NSSI methods was significantly related to both Negative and Positive Urgency (i.e., the tendency to act impulsively when experiencing negative/positive affect). This finding suggests that both negative and positive affects may trigger NSSI. There are a couple of potential explanations for this unique finding. First, some studies have found that the valence (positive/negative) of the emotion is not predictive of NSSI, but the intensity or variability of the emotions is significantly predictive of NSSI [20, 23, 24]. It may also be that the experience of positive emotions could be experienced as cognitive dissonance (e.g., I do not deserve to be happy), which may then trigger NSSI. This explanation is consistent with self-punishment models of NSSI [23]. It will be important for future studies to examine this idea as well as to continue to include positive affect when studying emotional correlates of NSSI [25]. The current finding that Positive/Negative Urgency positively correlated with the different NSSI behaviors affirms the affect-regulation functions of NSSI in general [23, 24]. For clinical practice, it seems advisable to help self-injurious students to find less harmful behaviors to deal with both, negative and positive affects.

Some additional interesting findings from the current study include the unique associations between select NSSI behaviors and the UPPS-P dimensions. For example, severe cutting was also significantly related to Lack of Premeditation (i.e., the tendency to act without consideration of the potential consequences of the behavior), but superficial cutting was not. It may be that individuals who do engage in some considerations of the potential consequences of cutting oneself are better able to regulate the severity of their cutting, and thus, may prevent some students form cutting too deep. Therefore, it may be useful for therapists that help individuals to actively consider the range of consequences of the NSSI (certainly in the presence of negative affect) to prevent more severe injuries or prevent NSSI altogether by means of self-regulation strategies. 

Additional findings from the current study include the unique associations between different NSSI functions and the UPPS-P dimensions. Students engaging in NSSI to regulate their emotions (F4) or to escape from others/reality/intrusions (F5) were characterized by impulsive actions when experiencing negative affect. This association may reflect high levels of emotional reactivity generally and possibly also in response to negative experiences. Thus, NSSI becomes a way to deal with negative affect and unwanted intrusions, which is consistent with prior studies [10]. Interestingly, the escape function of NSSI (F5) also related to positive urgency, and this could suggest a need to create or induce a more positive emotion quickly through the use of NSSI. Some research has indicated that NSSI is used to create a more positive emotional state (e.g., relief, calm) [20, 23, 24] as well as is associated with an inability to tolerate negative emotions [23, 24]. Additionally, students who engage in NSSI to approach/avoid destruction (F1) are characterized by a Sensation Seeking nature, that is the tendency to pursue actions that implicate a kind of “thrill,” which is consistent with some research indicating that particularly for males, NSSI is used to generate stimulation or experience “a rush” [4]. Finally, students who engage in NSSI to show that they are strong (F2) or to escape from school-work-related activities (F3) are characterized by Lack of Perseverance or the tendency to give up in the face of frustration. Maybe these students use their NSSI to compensate for potential feeling of failure or to have a way out of school-work-related activities because they experience them to be too difficult. Additional research should examine this unique finding. For therapists, it may be useful to consider the functions of NSSI in the view of the students' personality profile and find alternative behaviors which are in line with the students' self-reported NSSI functions and personality.

There are some limitations to the current study to consider. First, the study is based on self-report measures of both NSSI and personality; thus, shared method variance can partially explain the correlations between the variables. Second, the sample consisted of high-school students and needs further replication among patients with NSSI. Finally, the study is cross-sectional in nature, so future studies need to address the relationship between UPPS-P impulsivity traits and NSSI behaviors and functions in a longitudinal design to better understand the function of impulsivity in the onset and continuation of NSSI. Finally, based on the results of the present study, it is important not only to include measures of negative affectivity (e.g., anxiety, depression) while investigating NSSI, but also to pay attention to positive emotions when investigating NSSI and related functions.

## Figures and Tables

**Table 1:** Spearman's rank correlations between the UPPS-P impulsivity dimensions and the presence/absence of different NSSI methods (*N* = 613).

	Scratching	Superficial cutting	Severe cutting	Hitting	Burning	Head banging
Negative	.15**	.18**	.21**	.14**	.09*	.13**
Urgency						
Lack of Premeditation	−.02	−.06	.10*	−.03	−.01	.03
Lack of Perseverance	−.07	.02	.06	.01	.00	.07
Sensation	−.02	−.02	.03	.02	.01	.07
Seeking						
Positive	.09*	.11*	.09*	.11**	.04	.12**
Urgency						

*P < 0.05;  **P < 0.01.

**Table 2:** The factor loadings, means, and standard deviations of the NSSI functions, and the relationship between the NSSI function factors and the UPPS-P impulsivity dimensions.

	Factor loadings					*M* ^ a^	(SD)
	F1^b,c^	F2	F3	F4	F5		
To escape from a twilight or numb state	.92					1.30	(.73)
To avoid or suppress suicidal thoughts	.80					1.54	(.99)
To make myself unattractive	.71					1.15	(.61)
To show myself how strong I am		.86				1.52	(.87)
To show others how strong I am		.86				1.16	(.50)
To avoid school, work, or other activities			.79			1.10	(.36)
To avoid doing something unpleasant, you do not want to do			.78			1.11	(.43)
To punish myself				.86		2.19	(1.42)
To avoid or suppress negative feelings				.69		2.35	(1.32)
To avoid being with people					.89	1.10	(.49)
To get into a twilight or numb state					.56	1.39	(.79)
To avoid or suppress painful images or memories					.50	2.26	(1.44)

			Correlations				
	F1	F2	F3	F4	F5		

Negative Urgency	.20	.12	.06	.36**	.41**		
Lack of Premeditation	.13	.03	.10	.08	−.08		
Lack of Perseverance	.09	.19	.19	−.02	−.05		
Sensation Seeking	.24*	−.05	.16	−.09	.12		
Positive Urgency	.20	.17	.06	.16	.37**		

^a^Functions are assessed on a scale ranging from 1 (*not at all applicable*) to 5 (*very much applicable*).

^b^Only factor loadings >.40 are displayed.

^c^F1: avoid/approach destruction, F2: show being strong, F3: avoid tasks/events, F4: emotion regulation, F5: escape from others, reality, and intrusions.
